# The visual light field in real scenes

**DOI:** 10.1068/i0654

**Published:** 2014-11-28

**Authors:** Ling Xia, Sylvia C. Pont, Ingrid Heynderickx

**Affiliations:** π-lab (Perceptual Intelligence lab), Department of Industrial Design, Delft University of Technology, Delft, The Netherlands; e-mail: l.xia-1@tudelft.nl; π-lab (Perceptual Intelligence lab), Department of Industrial Design, Delft University of Technology, Delft, The Netherlands; e-mail: s.c.pont@tudelft.nl; Department of Human Technology Interaction, Eindhoven University of Technology, Eindhoven, The Netherlands; e-mail: i.e.j.heynderickx@tue.nl

**Keywords:** probing method, 3D texture, swap effect, direction, intensity, diffuseness

## Abstract

Human observers' ability to infer the light field in empty space is known as the “visual light field.” While most relevant studies were performed using images on computer screens, we investigate the visual light field in a real scene by using a novel experimental setup. A “probe” and a scene were mixed optically using a semitransparent mirror. Twenty participants were asked to judge whether the probe fitted the scene with regard to the illumination intensity, direction, and diffuseness. Both smooth and rough probes were used to test whether observers use the additional cues for the illumination direction and diffuseness provided by the 3D texture over the rough probe. The results confirmed that observers are sensitive to the intensity, direction, and diffuseness of the illumination also in real scenes. For some lighting combinations on scene and probe, the awareness of a mismatch between the probe and scene was found to depend on which lighting condition was on the scene and which on the probe, which we called the “swap effect.” For these cases, the observers judged the fit to be better if the average luminance of the visible parts of the probe was closer to the average luminance of the visible parts of the scene objects. The use of a rough instead of smooth probe was found to significantly improve observers' abilities to detect mismatches in lighting diffuseness and directions.

## 1 Introduction

The interplay between lighting, geometry, and materials in a scene shapes the architectural space and the light field in it. The structure of the light field depends on the spatial and spectral characteristics of the light sources, and on the shape, 3D texture (corrugation), and material reflectance properties of the patches in the light field that receive and re-emit the light. According to Gershun, if color and temporal variations are neglected, the light field is a 5-dimensional function that describes the light traveling in every direction (θ, φ) through any point (*x*,*y*,*z*) in space (Gershun, Moon, & Timoshenko, 1939). Gershun's light field is essentially the radiance distribution over space and directions. The theory of the light field developed by Gershun describes light physically (optically). It describes everything that can potentially be seen and in psychology this concept was called the “plenoptic function” by Adelson and Bergen (Adelson & Bergen, 1991).

Our interest mainly concerns the perception of the light field; are people able to discern all aspects of the optical light field? A detection study done by Ostrovsky (Ostrovsky, Cavanagh, & Sinha, 2005) showed that observers were remarkably insensitive to inconsistencies of illumination direction. They used both computer renderings consisting of identical but randomly oriented cubes and photographs of real scenes in which local inconsistencies of illumination were created. Their results suggest that the visual system does not verify the global consistency for locally derived estimates of illumination direction. However, Koenderink et al. found that human observers have expectations of how an object would look like when it was introduced at an arbitrary location in a scene (Koenderink, Pont, van Doorn, Kappers, & Todd, 2007). It means that human observers can infer the light field even in the empty space around them, which the authors called the “visual light field.” Later, Schirillo confirmed this theory on the basis of a review study (Schirillo, 2013). In this study, Schirillo presented both direct and indirect evidence of our awareness of the light field.

Which properties of the light field can human observers perceive? The light field can strongly influence the appearance of a scene and inversely, its properties can be reflected by the appearance of a scene. The light field in natural scenes is highly complicated due to intricate optical interactions, containing low and high frequencies in the radiance distribution function. Nonetheless, the human visual system (HVS) is able to distinguish the intensity, the primary illumination direction, and the diffuseness, which are basic (low-order) properties of a light field (Cuttle, 2003; Frandsen, 1989; Koenderink, van Doorn, Kappers, Pont, & Todd, 2005; Morgenstern, 2011; Morgenstern, Geisler, & Murray, 2014; Mury, Pont, & Koenderink, 2007; O'shea, Agrawala, & Banks, 2010; Pentland, 1982; Pont, 2013; Te Pas & Pont, 2005). Mury et al (Mury, 2009) described the relations between the mathematical structure and physical meaning of the light field components, plus a manner to measure and visualize the light field. The intensity corresponding to “radiant flux density” in Gershun's theory describes a constant illumination from all directions, which is usually known as “ambient light” in computer graphics. The “light vector” describes the average illumination direction and strength. Diffuseness of light was not mathematically described by Gershun or Mury and it ranges from fully collimated via hemispherical diffuse to completely diffuse light. An overcast sky generates hemispherical diffuse light, while direct sunlight is a typical example of collimated light and a polar whiteout of completely diffuse light.

The next question is how to reliably measure the perceived light field properties. Koenderink et al.'s experiment (Koenderink et al., 2007) adopted a method based upon the generic notion of a “gauge object.” Their stimuli were stereo photographs of a scene consisting of matte white painted penguins facing each other. A suitable “gauge object” was introduced at various locations in the photographic scenes and the observers were asked to adjust the appearance of the gauge object to visually fit the scene with respect to the lighting. Three properties of the light field (i.e., intensity, direction, and diffuseness) could be adjusted simultaneously. The results suggested that observers could accurately match the illumination at arbitrary locations in a static scene. The high reliability of the observers on their settings also demonstrated the validity of the method of using a “gauge object” to measure the visual fit with a scene. In our research, a similar experiment with the same method is conducted. However, instead of using photographs, we perform our experiment in a real scene by optically mixing a gauge object into a group of scene objects.

Looking at a real scene is totally different than looking at a picture of it, even when stereo pictures are used. There are a few reasons. First, there is a big difference in the dynamic range (DR) of luminance values between the real world and a computer screen. The real world has about 10 orders of dynamic range for luminance values spread across the spectrum from darkness to brightness while a computer screen has only approximately two to three (Myszkowski, Mantiuk, & Krawczyk, 2008). Second, some depth cues also induce differences between the real world and synthetic stereo images. The depth cue “motion parallax” is one of them. Observers can't stay absolutely still. When they move, the apparent relative motion of several stationary objects against a background gives hints about their relative distance, which can provide absolute depth information if the direction and velocity of the movement are known (Ferris, 1972). Besides head movements, eye movements can also provide additional information about the observed objects (Wismeijer, Erkelens, van Ee, & Wexler, 2010).

**Figure 1.**
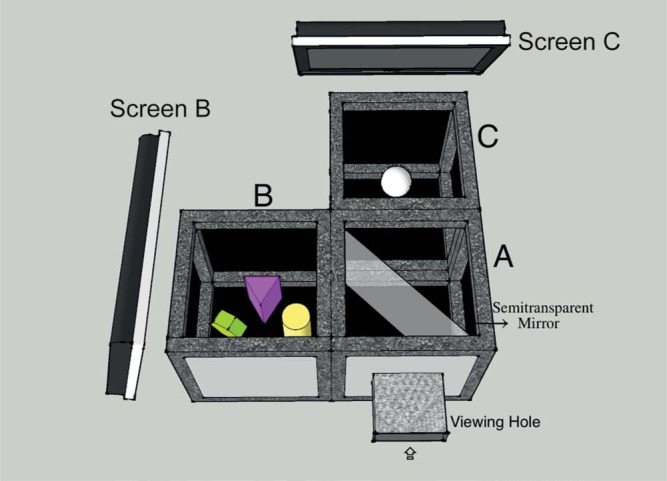
Illustration of the setup. The setup consists of three 30 cm × 30 cm × 30cm cubes, of which the inside is covered with black velvet paper. In cube B, we made a simple scene with five geometrical shapes. In cube C, we placed a white sphere, which served as the probe. A semitransparent mirror was placed vertically at the diagonal of box A. Two LCD screens, one covering the top of cube B and one the top of cube C, provided the lighting which could thus be varied independently for the scene and probe.

In this paper, we will implement a novel experimental setup to introduce a real gauge object into a real scene (Xia, Pont, & Heynderickx, 2013). A similar setup was first used in a material perception study in which real objects with different materials were optically mixed together (Pont, Koenderink, Van Doorn, Wijntjes, & Te Pas, 2012). The key point of this setup lies in the use of a semitransparent mirror. The scene and probe are located inside two separated boxes and they are mixed together optically by a semitransparent mirror (see Figure 1). In this way, the properties of lighting for the probe and scene can be changed separately without any mutual interference. We will focus on testing observers' sensitivities for illumination direction, diffuseness, and intensity because former studies into picture perception showed that observers are sensitive to these low-order characteristics of light. Observers have to judge the visual “fit” of the probe in the scene.

In the experiment, two types of probes will be used, a smooth sphere and a rough sphere (golf ball). Theoretically, the illuminance flow or texture contrast gradients over the sphere due to the roughness of the golf ball give cues about its illumination additional to shading (Koenderink & Pont, 2003; Pont & Koenderink, 2004). It has been found that observers are sensitive to such gradient orientations on flat rough surfaces (Koenderink et al., 2003), but to our knowledge this is the first systematic study of whether observers use such cues on 3D objects to judge specific illumination characteristics.

## 2 Experimental setup

**Figure 2.**
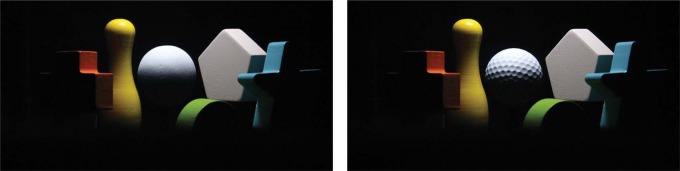
Optical mixture of scene and probe photographed from the viewing hole, with a smooth probe (left image), and a rough probe (right image).

We made a setup to optically mix the scene and probe object, as shown in Figure 1. Three 30 cm ×30 cm×30 cm cubes formed the main framework of this setup. In cube B, there were five colorful geometrical shapes forming a simple scene. In the center of cube C, there was a white sphere, which served as the probe. Because a white object has a higher albedo than an object with any other color, one of the colorful geometrical shapes was painted white to provide an anchor. Inside cube A, a semitransparent optical mirror was placed vertically at the diagonal, which was at 45º with respect to the viewing direction. Due to this mirror, the probe was seen through the mirror within the scene via its reflection in the mirror. The edges of the mirror could not be seen through the viewing hole. All cubes' insides were covered with light absorbing blackout material (black flocked paper, from Edmund Optics) to avoid too much scattering from the background, optical cross-talk through, and haze on the mirror. Occlusions between the probe and the shapes in the scene were prevented. Thus, when observers looked through the viewing hole, they saw the optical mixture of the scene and probe as if they were put together, as illustrated in Figure 2. The lighting of the scene and probe were provided by one LCD screen on top of cube B and one on top of cube C. Independent images were displayed on the two screens to determine the individual lighting for the scene and for the probe.

To make sure that both the probe and scene contributed 50% to the final superpositioned scene, we calibrated our setup. First, the relationship between the screens' luminance and their pixel values was measured. Then, we measured the reflectivity and transmission of the semitransparent mirror, being 41% and 59%, respectively (adding up to 100%, since this was a high-quality semitransparent mirror). By multiplying the luminance of screen B with the reflectivity of the mirror, we calculated the “simulated luminance” for screen B. In the same way, we calculated the “simulated luminance” for screen C by multiplying the luminance of screen C with the transmission of the mirror.

### 2.1 Lighting stimuli

**Figure 3.**
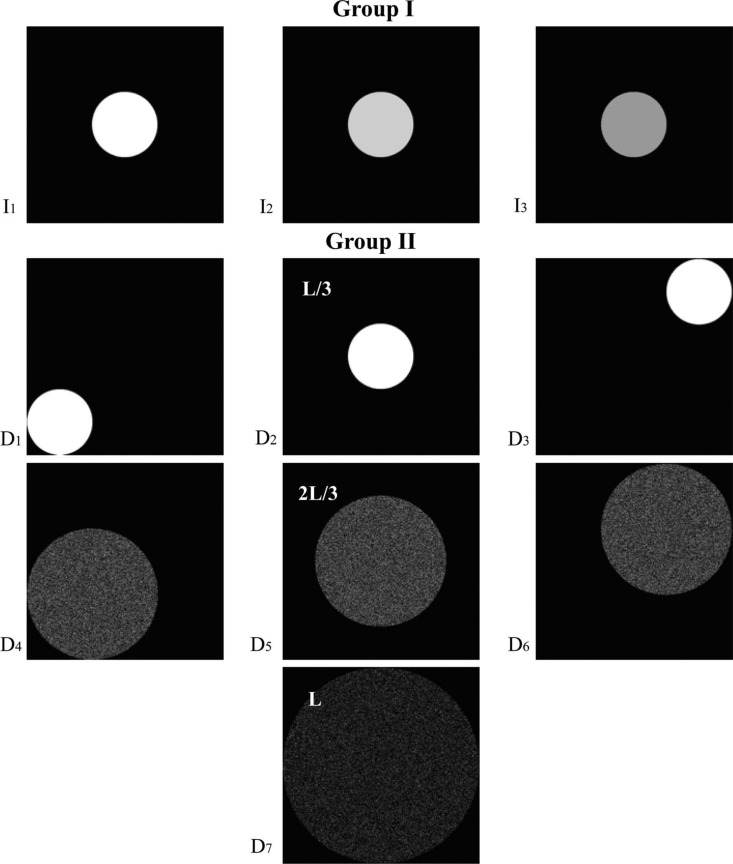
Lighting stimuli: Group I, variations in intensity which were achieved by changing the pixel values of the image of a disk (diameter of *L*/3, *L* = 25 cm) in the center of the LCD screen; Group II, variations in direction and diffuseness which were achieved by changing the position and the size of the displayed white disks. The disks were displayed in the center and in two opposite corners and had a diameter of *L*/3, 2*L*/3, and *L* (*L* = 25cm).

Two groups of images were used to simulate the light sources on the scene and probe, as depicted in Figure 3. The first group of stimuli was made to simulate intensity variations (group I) and a second group of stimuli was made to simulate variations in direction and diffuseness (group II).

In stimulus group I, we investigated whether human beings are sensitive to variations in lighting intensity over a scene. The lighting intensity variations were achieved by varying the pixel value of an image consisting of a white disk in the center of the LCD screen. The diameter of the white disk was *L*/3 (*L* = 25 cm, which was the width of the cube's top window). Three levels of intensity were used, marked as *I*_1_, *I*_2_, and *I*_3_ (with *I*_1_>*I*_2_> *I*_3_). For each level of intensity, we selected pixel values such that the “simulated luminance” of screen B and screen C were the same, as given in Table 1. It has long been known that the HVS has a nonlinear response to luminance. This nonlinearity can be quantified using the concept of JND (Just Noticeable Difference), which represents the luminance difference between two patches that the average human observer can just perceive under given viewing conditions. Following the definition, the difference between two luminance levels on the perceptual scale is proportional to the number of JNDs between them (Xu, Saha, Guarnieri, Ramponi, & Badano, 2008). Based on the Barten model (Barten, 1992; Barten, 1993), Rosslyn et al (NEMA, 2000) developed a Grayscale Standard Display Function which describes the relationship between luminance and Just-Noticeable Differences. When selecting three intensity levels, we made sure that the difference in luminance between *I*_1_ and *I*_2_, and between *I*_2_ and *I*_3_ had the same number of JNDs. The *I*_1_ and *I*_3_ were selected such that the difference when *I*_1_ illuminated the scene and *I*_3_ illuminated the probe, or vice versa, was obvious (based on a pilot experiment with seven observers). The difference in luminance between *I*_1_ and *I*_2_, and between *I*_2_ and *I*_3_ were 60 JNDs.

**Table 1.** Pixel values, simulated luminance values, and their corresponding JND levels for three levels of intensity

	*I*_1_	*I*_2_	*I*_3_
Pixel values of screen B	255	135	85
Pixel values of screen C	156	103	68
Simulated luminance (cd/m^2^)	77	47	28
JND values	441	381	321

In stimulus group II, we varied the position and the size of the disk to investigate whether human beings are sensitive to the average direction as defined by the light vector (Gershun et al., 1939; Mury, 2009; Mury et al., 2007) and to the diffuseness as defined by the scale of light of a light source (Frandsen, 1989). We displayed white disks with a diameter of *L*/3 in the center and in two opposite corners of the screen to vary the direction, and displayed white disks with diameters *L*/3, 2*L*/3, and *L* to investigate the sensitivity to diffuseness. The simulated luminance for screen B and screen C was set to their maximum of 77 cd/m^2^, corresponding to *I*_1_. As we needed to keep the total emitted luminous flux for the three diameters of the white disk the same, we used the same number of white pixels as for *L*/3, but randomly distributed them over the disk when its diameter was larger than *L*/3. This created a noise pattern in the LCD image mimicking the light source (as shown in Figure 3), but didn't impact the light on the probe or scene objects. We also varied the position of the disk for the diameters *L*/3 and 2*L*/3 to investigate the interaction between direction and diffuseness. We refer to the stimuli in Group II by D_1_ to D_7_.

### 2.2 Probe

This experiment was performed with two different probe objects, i.e., a smooth sphere and a golf ball (see Figure 2), in order to check whether human beings are sensitive to illuminance flow over a rough probe. The dimples on the golf ball resulted in 3D texture contrast gradients over the ball, which generated “illuminance flow” and provided cues additional to the shading.

### 2.3 Task

The task of the observer was to judge whether the white probe fitted the scene with regard to its lighting. If the observer thought the lighting of the scene and probe was the same, they had to press “Yes” on the keyboard. Otherwise, they had to press “No.” After they had given their answer, they had to press “confirm” to proceed to the next trial.

As illustrated in Figure 3, stimulus group I consisted of three lighting conditions, and so, resulted in nine combinations for scene and probe. Stimulus group II consisted of seven lighting conditions, and so, resulted in 49 combinations for scene and probe. So, among the total of 58 combinations, 48 had different lighting settings for scene and probe and 10 had the same lighting settings for scene and probe. We repeated the 10 “same combinations” for three times to improve the balance between the number of same and different combinations, resulting in a total of 78 comparisons to be made for one probe. As we used two probes, this resulted in 156 trials in total.

Before the formal experiment started, all participants were asked to do a few practice trials to become familiar with the procedure. The whole experiment was divided into two sessions, where a different type of probe was evaluated in each session. Since only one type of scene layout was used, there could be learning effects. In order to minimize the effects on the outcome, all stimuli were randomly given within each session and the order of the two sessions was balanced across participants. Between the first and second session, the observers took a break. The whole experiment took about half an hour for each participant.

### 2.4 Participants

Twenty observers participated in this experiment, 11 females and nine males, two of whom were the first and second author. Observers ranged in age from 24 to 43 and the median age was 30. The participants were naive with respect to the setup of this experiment except for the two authors. All participants had normal or corrected-to-normal vision. They all gave written, informed consent. All experiments were done in agreement with local ethical guidelines, Dutch Law, and the Declaration of Helsinki.

## 3 Results

This results section is organized as follows: first, we analyze the overall percentages of correct answers; after that we use the data of Group I to investigate how sensitive our observers are to variations in lighting intensity; then the data of Group II are used to check how sensitive they are to the variations of lighting direction and diffuseness. For all statistical tests applied below, we measured the significance at the 0.05 level.

When examining the overall percentages of correct answers, we distinguish two options: for the stimuli with the same lighting on probe and scene, “Yes” was the correct answer, while for the stimuli with different settings, “No” was the correct answer. Consequently, we split the data into two categories, one category “stimuli with same lighting” and another category “stimuli with different lighting.” The percentages of correct answers are given in Table 2. We used one-sample binomial tests to check whether these percentages were significantly different from 50%. The results showed that they all were significantly different from chance level.

**Table 2.** Percentages of correct answers split up for stimuli with the same lighting setting on scene and probe and stimuli with different lighting setting on scene and probe (*N* = total number of answers, Percentage = percentage of correct answers)

		Stimuli with same lighting		Stimuli with different lighting	
Stimuli	Type of probe	N	Percentage	N	Percentage
Group I	Smooth	180	77%	120	66%
	Rough	180	73%	120	67%
Group II	Smooth	420	80%	840	61%
	Rough	420	78%	840	65%

**Figure 4.**
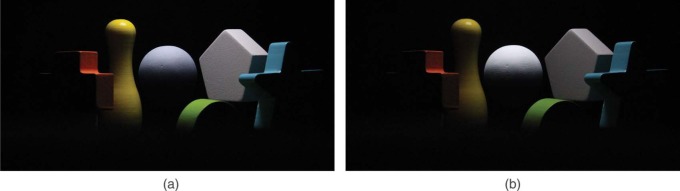
“Swap effect” for lighting settings with different intensity: (a) lower intensity on the probe and higher intensity on the scene (percentage of correct answers: 35%), (b) lower intensity on the scene and higher intensity on the probe (percentage of correct answers: 75%)

**Figure 5.**
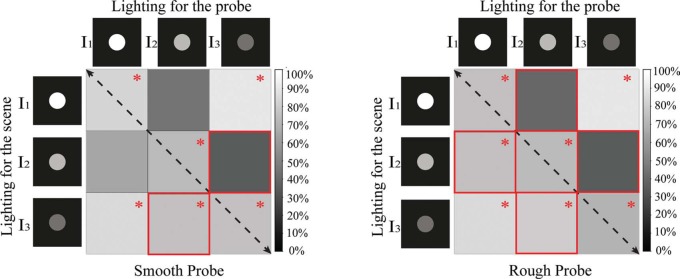
Matrix graphs representing the percentages of correct answers for stimulus group I. The columns represent the intensity level on the probe, while the rows represent the intensity level on the scene. The gray level indicates the percentage of right answers. Black means that 0% of the answers was correct. For the cells marked with a red “*,”the proportion of correct answers was significantly different from 50%, according to the result of a one-sample binomial test. The paired cells marked with a red solid frame show where the swap effect occurred according to a Pearson's Chi-square test. The left graph represents the data for the smooth probe and the right graph the data for the rough probe.

**Figure 6.**
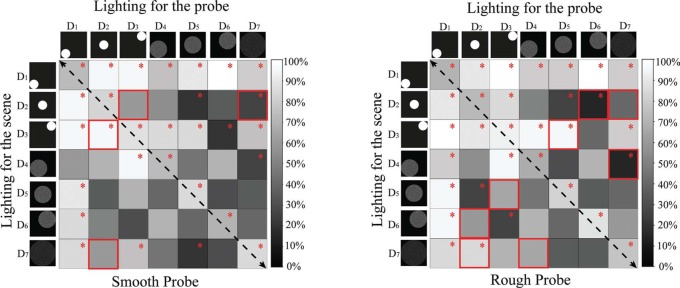
Matrix graphs representing the percentages of correct answers for stimulus group II. The columns represent the lighting setting on the probe, while the rows represent the lighting settings on the scene. The gray level indicates the percentage of correct answers. Black means that 0% of the answers was correct. For the cells marked with a red “*,” the proportion of correct answers was significantly different from 50%, according to the result of a one-sample binomial test. The paired cells marked with a red solid frame show where the swap effect occurred according to a Pearson's Chi-square test. The left graph shows the data for the smooth probe and the right graph the data for the rough probe.

Before we go into a more detailed analysis, we introduce the phenomenon of the “swap effect.” For some combinations of the scene and probe lighting, participants judged the probe to fit the scene well, yet swapping the lighting over the probe with that over the scene dramatically shifted participants' judgments. One example is given in Figure 4, where the optical mixture of a lower intensity *I*_3_ on the probe and a higher intensity *I*_2_ on the scene (Figure 4(a)) looks as a good fit between probe and scene (only 35% of the observers recognized a mismatch between probe and scene), while the optical mixture of a higher intensity *I*_2_ on the probe and a lower intensity *I*_3_ on the scene (Figure 4(b)) does not look as a good fit between probe and scene (indeed 75% of the observers recognized the mismatch in illumination on probe and scene). Because of this asymmetry, we take this “swap effect” into consideration in the rest of our analysis. To quantify the combinations of lighting on probe and scene as susceptible to the “swap effect,” we used the Pearson's Chi-square test on the percentages of correct answers for the combinations of mirrored lighting on probe and scene. The lighting combinations that resulted in a significant difference are marked with a red solid frame in the matrix graphs of Figures 5, 6, 8, and 10.

### 3.1 Group I results: intensity sensitivity

The percentages of correct answers to compare lighting intensity on probe and scene are visualized in Figure 5 for each possible combination. The gray level indicates the percentage of correct answers, where black means that 0% of answers is correct. One-sample binomial tests were performed to check whether the percentage of right answers was significantly different from chance. Cells in the matrix graph of Figure 5 for which this was the case are marked with red “*.”

We noticed that the data in Figure 5 are not symmetrical with respect to the black dashed diagonal (from upper left to bottom right), which is due to the “swap effect.” The lighting combinations where the “swap effect” occurred are marked with a red solid frame. The cells in the upper right represent lighting with lower intensity on the probe than on the scene (see Figure 4(a)). The cells in the bottom left represent lower intensity on the scene than on the probe (see Figure 4(b)). The combination “*I*_1_∼*I*_3_” had the largest difference in intensity (120 JNDs), and the observers were quite sensitive to it with more than 85% of correct answers. For the combinations “*I*_1_∼*I*_2_” and “*I*_2_∼*I*_3_”, the difference in intensity was much smaller (60 JNDs), but still rather well perceived if the lower intensity illuminated the scene and the higher intensity illuminated the probe (except for the combination “*I*_1_∼*I*_2_” for the smooth probe). However, when the lower intensity illuminated the probe and the higher intensity illuminated the scene, the observers had difficulties seeing the mismatch (the percentage of correct answers was not significantly different from the chance level).

We employed a binary logistic analysis of the generalized linear model to analyze the probability that the observers could see the difference in lighting intensity between probe and scene. Only the combinations with different intensity levels of lighting for scene and probe were included in this analysis. The predictable variables were the type of probe, the “swap effect,” and the difference in intensity level. The “swap effect” included two cases: (1) lower intensity on the probe than on the scene, and (2) lower intensity on the scene than on the probe. Two-way interactions between probe type and intensity difference and between the “swap effect” and intensity difference were also included. The results are given in Table 3. There was no significant difference in percentage of correct answers between the smooth and rough probe. Also, the interaction between probe type and intensity difference was not significant. The influence of the “swap effect” and intensity were both statistically significant (*p*<0.05), as well as their interaction. The latter is consistent with what we described before: people are sensitive to a small difference in intensity only when the scene is illuminated by the lower intensity and the probe by the higher intensity, but this “swap effect” disappears when the intensity difference is large enough.

**Table 3.** Results of a binary logistic analysis for the effect of probe type, “swap effect,” and intensity difference on the percentage correct answers

Source	Wald Chi-square	Df	P
Probe type	0.059	1	0.808
Swap effect	6.826	1	0.009
Intensity difference	18.994	2	<0.001
Probe type*intensity difference	0.024	2	0.988
Swap effect* intensity difference	7.426	2	0.024

### 3.2 Group II result: direction and diffuseness sensitivity

To give an overview of the data, the percentages of correct answers for different combinations of direction and diffuseness are visualized as matrix graphs in Figure 6. Again, the gray level in the cell represents the percentage of correct answers, where black means that 0% of the answers was correct. In Figure 6, it can be seen that the global patterns for the performance as a function of lighting direction and diffuseness are quite robust. In both graphs, the grey levels are not symmetrically distributed with respect to the diagonal (from upper left to bottom right) due to the “swap effect.” Where it occurs, the corresponding cells are marked with a red solid frame.

**Figure 7.**
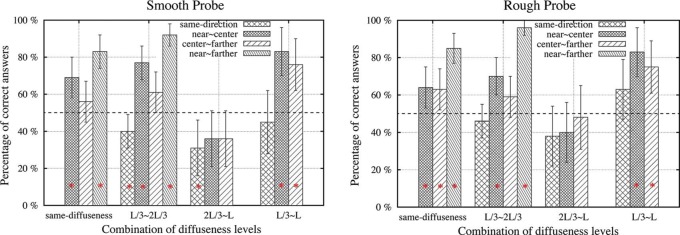
Percentages of correct answers as a function of the difference in diffuseness for different pairs of directions. The left graph represents the data for the smooth probe and the right graph for the rough probe. The bars marked with red “*” had a percentage of correct answers significantly different from 50%. Error bars show the 95% confidence interval.

For further analysis, Figure 7 illustrates the percentages of correct answers for lighting with different directions or different diffuseness or both, for the smooth probe and the rough probe, respectively. We did not consider the “swap effect” in Figure 7 because the “swap effect” for directions differs from that for diffuseness. Later, we will discuss the “swap effect” for direction and diffuseness separately. The bars labeled as “same-direction” represent cases with the same direction on the probe and the scene. Since only the data for combinations with different lighting settings for scene and probe were considered here, the bar labeled as “same-direction” was absent from the diffuseness combination of “same-diffuseness” in Figure 7. Likewise, because the display disk with the largest diffuseness *L* could only be located in the center, the bars labeled as “near ∼farther,” which represent direction variation from the near corner to far corner, were absent from the diffuseness combinations “2*L*/3∼*L*” and “*L*/3∼*L*.” One-sample binomial tests were conducted to evaluate to what extent the percentages correct answers differed from a chance level of 50%. If the latter was the case, the corresponding bar in Figure 7 is marked with a red “*.” If the percentage was significantly lower than the chance level, it indicated that the observers consistently saw the lighting on the scene and probe to fit. If the percentage was significantly higher than the chance level, it implied that the observers had no difficulty to perceive the mismatch in lighting on scene and probe.

Again, we used a binary logistic analysis of a generalized linear model to analyze the probability that the observers could see the mismatch in lighting for the probe and the scene. The predictable variables were the type of probe, the difference in direction, and the difference in diffuseness. All two-way interactions were included. Since the “swap effect” for direction and diffuseness was different and will be discussed separately later, we did not introduce the “swap effect” into this analysis. The results in Table 4 reveal that the influence of probe type was statistically significant (*P*<0.05). Thus, as we expected, the probe type did have an effect on the detection of combined direction and diffuseness differences while it had no effect on the detection of intensity differences. Overall, the percentage of correct answers was higher for the rough probe than for the smooth probe. The effects of differences in direction and in diffuseness were both highly significant. The first set of bars in Figure 7 (i.e., differences in direction for the same diffuseness) illustrates that as the difference in direction increased, more and more people could see that the directions did not fit. Generally, as the difference in diffuseness became larger (i.e., illustrated by the same type of bars in different sets in Figure 7), more of the observers could detect the mismatch. The percentage of correct answers was especially low for the combination “2*L*/3∼*L*.” We also found a significant interaction between direction and diffuseness differences. These results were in agreement with what Figure 7 showed. In general, observers were very sensitive to differences in lighting direction, but this sensitivity was affected by the lighting diffuseness. When the diffuseness was relatively large, but different for probe and scene *(*i.e., for the combination *2L*/*3∼L),* most of the observers tended to be uncertain whether the scene and probe fit each other (since both in Figure 7(a) for the smooth probe and in Figure 7(b) for the rough probe most of the bars were not significantly different from chance level).

**Table 4.** Results of a binary logistic analysis for sensitivity to differences in lighting direction and diffuseness, including the effect of probe type

Source	Wald Chi-square	df	P
Probe type	3.963	1	0.047
direction difference	103.719	3	<0.001
diffuseness difference	60.926	3	<0.001
direction difference*diffuseness difference	14.166	6	0.028
Probe type*direction difference	4.258	3	0.235
Probe type*diffuseness difference	1.957	3	0.581

#### 3.2.1 Swap effect

The influence of the “swap effect” for direction and diffuseness was analyzed separately to investigate what happened when the lighting with only different directions or only different diffuseness was swapped between probe and scene. We selected the data with only one kind of difference either in direction or in diffuseness, to avoid mutual impact. The “swap effect” for direction included two conditions: (1) farther lighting on the probe and closer on the scene, and (2) farther lighting on the scene and closer on the probe. Similarly, the “swap effect” for diffuseness included also two conditions: (1) larger diffuseness on the probe and smaller on the scene, and (2) larger diffuseness on the scene and smaller on the probe.

**Figure 8.**
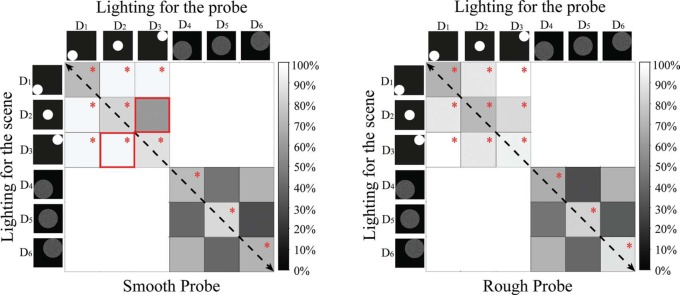
Matrix graphs representing the percentages of correct answers for combinations of different directions but same diffuseness on the probe and the scene. The columns represent the lighting condition on the probe, while the rows represent the lighting condition on the scene. The gray level in each cell indicates the percentage of correct answers. Black means that 0% of the answers was correct. The cells marked with red “*” indicate that the proportion of correct answers for this combination of lighting on probe and scene was significantly different from 50%. The paired cells marked with a red solid frame show where the swap effect occurred according to a Pearson's Chi-square test. The left graph represents the data for the smooth probe and the right graph for the rough probe.

For lighting settings with different directions but same diffuseness, both stimuli with diffuseness of *L*/3 and 2*L*/3 were selected as shown in Figure 8. The cells right above the black dashed diagonal (from upper left to bottom right) in the matrix graph represent farther lighting on the probe than on the scene, while the cells left below the diagonal represent farther lighting on the scene than on the probe.

**Figure 9.**
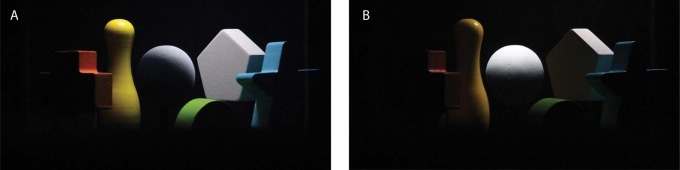
Optical mixture of scene with center lighting (D_2_) and right far lighting (D_3_): The left image represents center lighting on the scene and right far lighting on the probe; The right image represents center lighting on the probe and right far lighting on the scene.

Generally speaking, both graphs in Figure 8 were symmetrically mirrored with respect to the black dashed diagonal except for the combination D_2_-D_3_ when a smooth probe was used (as marked with a red solid frame in Figure 8 left graph). The result of a binomial test showed that when D_3_ was on the probe and D_2_ on the scene (as illustrated in the left image of Figure 9), the observers were just guessing whether both lighting conditions fitted. However, when D_3_ and D_2_ were swapped (as illustrated in the right image of Figure 9), the observers were quite sure that the light on the probe did not fit the light on the scene. We have no specific explanation for this observation, but just noticed (as can be seen from Figure 9) that the mixed scene looked more disharmonious with a brighter illumination on the probe when the center lighting was on the probe and the far lighting on the scene. A binary logistic analysis of a generalized linear model was performed using the probe type, the “swap effect,” the difference in direction, as well as their two-way interactions as predictor variables. However, the result showed that the “swap effect” did not statistically significantly influence observers' awareness of the mismatches the probe and scene (Chi^2^ = 1.179, *p* = 0.278).

**Figure 10.**
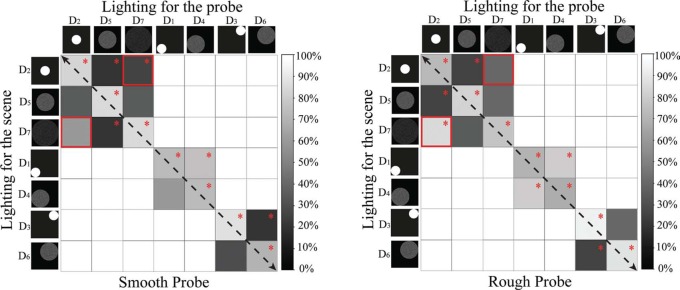
Matrix graphs representing the percentages of correct answers for combinations of different diffuseness but the same direction on the probe and the scene. The columns represent the lighting condition on the probe, while the rows represent the lighting condition on the scene. The gray value in each cell indicates the percentage of the correct answers. Black means that 0% of the answers was correct. The cells marked with red “*” indicate that the proportion of correct answers for this combination of lightings was significantly different from 50%. The paired cells marked with a red solid frame show where the swap effect occurred according to a Pearson's Chi-square test. The left graph represents the data for the smooth probe and the right graph for the rough probe.

For lighting settings with different diffuseness but same direction on scene and probe, stimuli in the center, in the near left corner, and in the far right corner were selected as shown in Figure 10. In each group of cells, the ones right above the black dashed diagonal (from upper left to bottom right) have a higher diffuseness on the probe than on the scene, while the ones left below the black dashed diagonal have higher diffuseness on the scene than on the probe. In general, each group of cells is pretty well mirrored with respect to its diagonal, suggesting that the “swap effect” was small, except for the combination D_2_-D_7_. Again, a binary logistic analysis of a generalized linear model was performed, using the probe type, “swap effect,” and difference in diffuseness as main predictor variables, including their two-way interactions. We did not find a significant main effect of the “swap effect” (Chi^2^ = 2.991, *p* = 0.084), but we did find a significant interaction between “swap effect” and “difference in diffuseness” (Chi^2^ = 12.432, *p* = 0.002). The latter was already suggested by our observations in Figure 10. With small “difference in diffuseness” between the probe and scene (*L*/3∼2*L*/3:D_2_-D_5_, D_1_-D_4_, D_3_-D_6_; 2*L*/3∼*L*:D_5_-D_7_), observers often had difficulties to see the mismatch in illumination diffuseness and there was no significant “swap effect”. However, when the “difference in diffuseness” was relatively large (*L*/3∼*L*:D_2_-D_7_), the “swap effect” was significant. In the latter case, the percentage of correct answers was higher when the lighting on the scene was more diffuse than on the probe, while the percentage of correct answers was much lower when the lighting on the probe was more diffuse than on the scene.

## 4 Discussion

We have used a novel experimental setup to investigate human sensitivity for lighting intensity, direction, and diffuseness in real scenes. One advantage of this setup is that it allows us to introduce a real probe object into a real scene while the lighting of the scene and the probe can be varied separately without any mutual interference. Furthermore, the task to judge the visual “fit” of the illuminated probe in a scene allows us to obtain a direct operational measure, which quantifies what the observer sees with a purely visual “yardstick” (Koenderink et al., 2007; Koenderink, van Doorn, & Wagemans, 2012). During the experiment, observers had little trouble with the task and none of them doubted that the probe and scene were actually not located in the same box.

Where previous studies (Koenderink et al., 2005, 2007; Pont & Koenderink, 2007) asked participants to adjust the lighting on the (virtual) probe such that it fitted into the (virtual) scene, we here simplified the task by asking observers only to indicate (with “yes” or “no”) whether the lighting of the probe fitted the scene. We repeated the stimuli with the same lighting on probe and scene three times each, so that the number of same combinations was 60, and the number of different combinations was 96 (see also Section 2). Table 2, however, shows that the number of “yes” and “no” responses was balanced. Indeed, for stimuli with the same lighting conditions (*N* = 60) participants on average had 78% correct answers (and so, on average 47 “yes” and 13 “no” responses), whereas for stimuli with different lighting conditions (*N* = 96) participants on average had 63% correct answers (and so, on average 36 “yes” and 60 “no” responses). So, the sum of the “yes” responses and “no” responses was much more balanced than what could be expected from the number of stimuli with the same or different lighting conditions. We don't think that this tendency of balancing the “yes” and “no” responses has influenced our findings, since it in principle affected all stimuli in a similar way.

We found that the observers were sensitive to variations of the light intensity, direction, and diffuseness, which confirmed the results of former studies. A study conducted by Pont et al. found that observers were able to match the direction and diffuseness of a light field (Pont & Koenderink, 2007). The visual light field study by Koenderink et al. certified observers' ability on estimating the direction and diffuseness in natural scenes and found that the observers were also sensitive to the variations in light field intensity (Koenderink et al., 2007). While their studies were all performed based on the use of images on computer screens, this study was conducted in real scenes. Hence, this study confirmed that observers' sensitivity to intensity, direction, and diffuseness of the light field also holds for real scenes. As expected, we found that as the difference in intensity, direction, and diffuseness increased, the observers were better aware of the mismatches between the scene and probe. Since the methods used in the former studies (adjustment to create visual fit) and in the current study (yes/no to visual fit) are so different, it is not possible to compare the results in terms of sensitivity.

It should be noted that the current study only used one particular, relatively simple real scene to demonstrate the measurement of the visual light field in a real scene. As participants assessed 156 stimuli with this particular scene, we cannot exclude a learning or adaptation effect to this scene. However, all stimuli in the experiment were provided to each participant in a different random order, and so, possible learning/adaptation to the stimuli is not expected to affect our findings. Obviously, more scenes need to be investigated to evaluate the generalizability of our findings to other scenes. We do expect that also in other real scenes people will be sensitive to the intensity, direction, and diffuseness of the light field, though possibly with different sensitivity.

**Figure 11.**
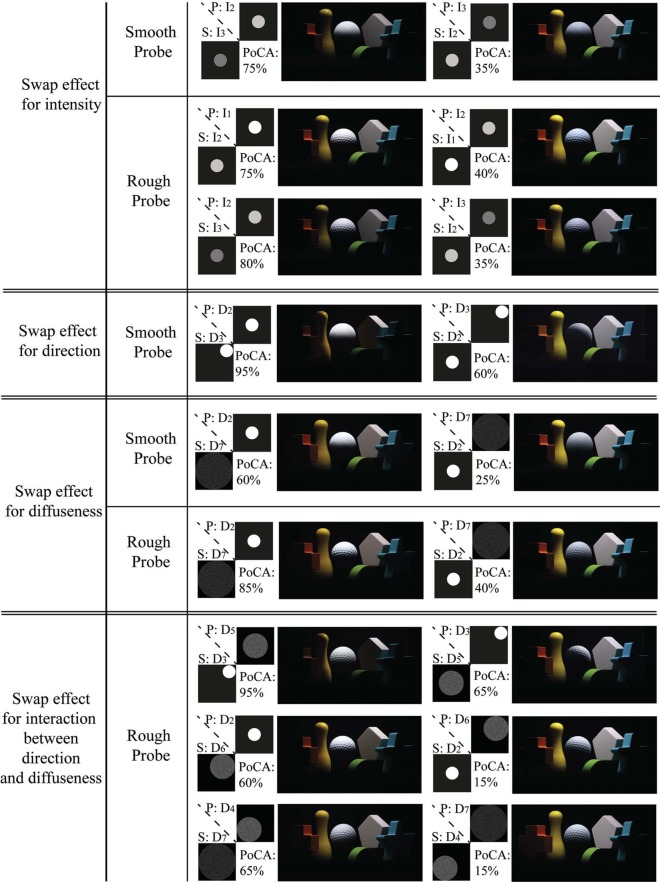
Optical mixtures of scene and probe for all the cases where the “swap effect” occurred. The lighting on the probe was marked as “P” (i.e., P:*I*_2_ means the probe was illuminated with intensity *I*_2_) and the lighting on the scene was marked as “S.” “PoCA” represents the percentage of correct answers. The higher the PoCA is, the more observers were aware of the “mismatch in lighting between scene and probe.”

We discovered in some cases a “swap effect,” which means that whether the mismatch in lighting between probe and scene was visible dependent on which of the two lighting conditions was on the scene and which on the probe. We listed the optical mixtures for all the cases where the “swap effect” occurred in Figure 11. For lighting conditions differing in intensity and diffuseness, the “swap effect” occurred with both the smooth probe and rough probe. The “swap effect” for one of the combinations of different directions of the lighting only occurred with the smooth probe.

**Figure 12.**
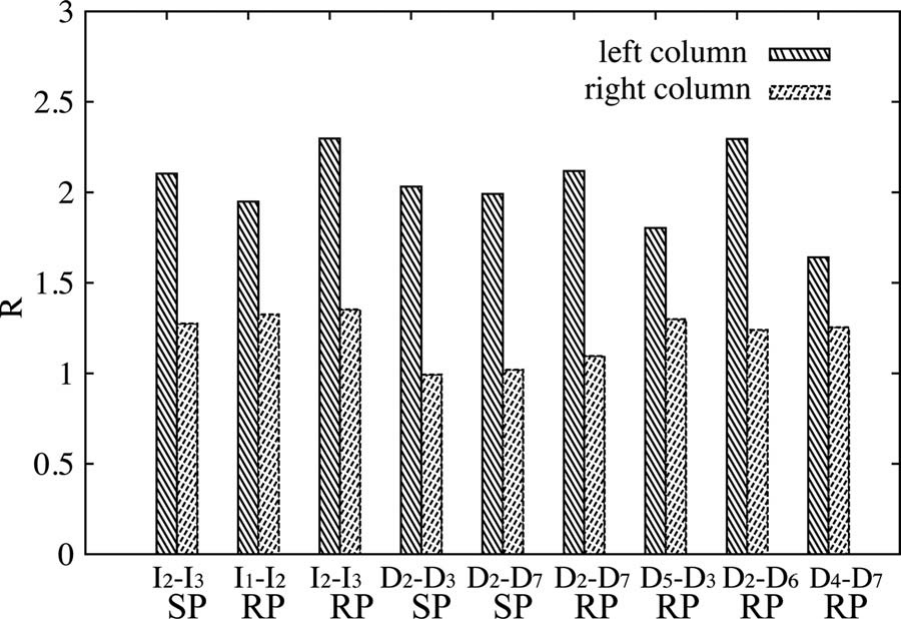
The ratio between the average luminance of the probe and the average luminance of the scene (*R* = Lum_probe/Lum_scene) for all the lighting combinations where the “swap effect” occurred. The bars labeled as “left column” and “right column” represent the optical mixtures in the left column and right column of Figure 11, respectively. The label SP represents results for the smooth probe and RP for the rough probe.

To investigate why the “swap effect” happened, we examined all optical mixtures for which it happened in Figure 11. The observers were significantly more aware of the mismatch between scene and probe for the conditions in the left column than for those in the right column according to the percentage of correct answers (PoCA). We noticed that the probes in the left column seemed to be brighter with respect to their surrounding scene than in the right column. Therefore we calculated the ratios between the average luminance of the probe and the average luminance of the scene (*R* = Lum_probe/Lum_scene) for the images of the optical mixtures in both the left and right columns, see Figure 12. Since we took the photographs using the same setting of the camera, the average luminance could be estimated via the average pixel luminance. To exclude the dark areas and noise in the photographs, only the pixels whose luminance was larger than 0.1 were considered. Figure 12 shows that the luminance of the probe with respect to the scene was indeed higher for all the optical mixtures in the left column. These results indicate that indeed the observers might have judged the mixes in the right column as a better fit because the average luminance of the visible parts of the probe were closer to the average luminance of the visible parts of the scene objects whereas in the left column the probe is clearly much higher in luminance.

**Figure 13.**
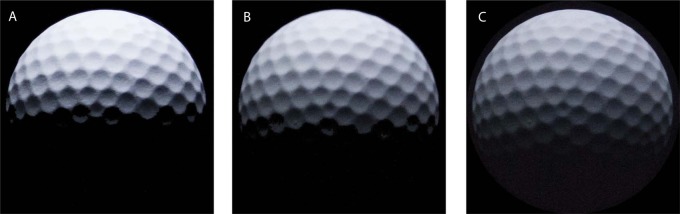
The illumination over the probe under different levels of diffuseness, the diameter of the disk being *L*/3 (8 cm) in the left image, 2*L*/3 (16 cm) in the middle image, and *L* (24 cm) in the right image.

In our experiment, the mean illuminance over the probe decreased as the diffuseness increased. Figure 13 illustrates the illumination over the probe under three diffuseness levels: from left to right, the diameter of the disk changes from *L*/3 (8 cm) to 2*L*/3 (16 cm) and *L* (25 cm), and the corresponding scale of light is 24%, 43%, and 59% according to the definition by Frandsen (Frandsen, 1989); so, the mean illuminance over the probe decreases by a factor of 92% and 83% in the middle and right image in comparison to the left image. This observation triggered another question, namely whether the observers detected the difference of diffuseness by just comparing the mean illumination on the probe and the scene. If so, they would have difficulties in judging where the inhomogeneity originated from: from the difference in intensity or from the difference in diffuseness, not to mention estimating the diffuseness and intensity simultaneously. Therefore, our next study will focus on whether observers can simultaneously match the intensity, direction, and diffuseness in real scenes.

Finally, our findings show that the use of a rough probe did not help the observers to detect the mismatch in intensity of the lighting between the scene and probe, but it helped them to find the mismatch in the combination of lighting direction and diffuseness between scene and probe. This proved our expectation that for real 3D objects, human observers can use the illuminance flow to judge illumination properties. In computational vision, the illuminance flow is derived from the roughness on the mesoscale. The variation of illuminance on the macroscale is usually denoted as “shading” and the variation of illuminance on the mesoscale as “3D texture” (Pont & Koenderink, 2004). In theory, the illuminance flow generated by 3D texture gradients can give additional cues about the illumination to the “shading” (Koenderink & Pont, 2003). This theory was confirmed for flat surfaces by asking subjects to adjust the elevation and azimuth of a probe for a series of pictures of natural textures (Koenderink, Doorn, Kappers, Pas, & Pont, 2003). In our experiment, the illuminance flow or texture contrast gradients over the rough probe give information about the direction and diffuseness. However, it doesn't give information about the intensity because the texture gradient structure is invariant for intensity changes. So, as expected, the 3D texture on the rough probe did not improve judgments of the mismatches in lighting intensity, but it did for diffuseness and direction. To our knowledge, this is the first systematic study proving that observers use the illuminance flow over 3D objects to judge specific illumination characteristics.

## 5 Conclusions

In summary, we implemented a novel experimental setup to investigate human sensitivity to low-order properties of light fields in real scenes, namely the intensity, direction, and diffuseness. We found that observers are able to detect whether a probe fits a scene with respect to these light qualities. However, in some cases with different lighting on the probe and scene, the awareness of the mismatch depended on which lighting condition was on the scene and which on the probe, which we called the “swap effect.” For these cases, we found that observers judged the fit to be better if the average luminance of the visible parts of the probe were closer to the average luminance of the visible parts of the scene objects. Finally, adding roughness to the spherical probe improved judgments of the mismatch in direction and the diffuseness of the lighting, showing that human observers use illuminance flow as a cue to the light.
